# NK Cells from RAG- or DCLRE1C-Deficient Patients Inhibit HCMV

**DOI:** 10.3390/microorganisms7110546

**Published:** 2019-11-10

**Authors:** Zeguang Wu, Narmadha Subramanian, Eva-Maria Jacobsen, Kerstin Laib Sampaio, Johannes van der Merwe, Manfred Hönig, Thomas Mertens

**Affiliations:** 1 Institute of Virology, Ulm University Medical Center, D-89081 Ulm, Germany; 2 Department of Pediatrics and Adolescent Medicine, Ulm University Medical Center, D-89081 Ulm, Germany; 3 Institute of Molecular Virology, Ulm University Medical Center, D-89081 Ulm, Germany

**Keywords:** human cytomegalovirus, natural killer cell, severe combined immunodeficiency

## Abstract

The recombination-activating genes (RAGs) and the DNA cross-link repair 1C gene (DCLRE1C) encode the enzymes RAG1, RAG2 and Artemis. They are critical components of the V(D)J recombination machinery. V(D)J recombination is well known as a prerequisite for the development and antigen diversity of T and B cells. New findings suggested that RAG deficiency impacts the cellular fitness and function of murine NK cells. It is not known whether NK cells from severe combined immunodeficiency (SCID) patients with defective RAGs or DCLRE1C (RAGs^−^/DCLRE1C^−^-NK) are active against virus infections. Here, we evaluated the anti-HCMV activity of RAGs^−^/DCLRE1C^−^-NK cells. NK cells from six SCID patients were functional in inhibiting HCMV transmission between cells in vitro. We also investigated the expansion of HCMV-induced NK cell subset in the RAG- or DCLRE1C-deficient patients. A dynamic expansion of NKG2C^+^ NK cells in one RAG-2-deficient patient was observed post HCMV acute infection. Our study firstly reveals the antiviral activity of human RAGs^−^/ DCLRE1C^−^-NK cells.

## 1. Introduction

HCMV is a ubiquitous herpes virus that persists in the host. Control of HCMV requires a continuous immune surveillance, including innate and adaptive immunity. Thus, HCMV is responsible for a high morbidity and mortality in immunocompromised patients [1], for example SCID patients whose immunity is impaired due to various genetic disorders.

T and B cells express a large repertoire of antigen-specific receptors that are generated by V(D)J somatic recombination. This process insures the highly diverse repertoire of immunoglobulins and TCRs and is mediated by V(D)J recombination machinery. The key components involved are RAG1 and 2, terminal deoxynucleotidyl transferase, Artemis nuclease, DNA-dependent protein kinase, X-ray repair cross-complementing protein 4, DNA ligase IV, non-homologous end-joining factor 1 and DNA polymerases λ and μ [2,3]. Mutations in these genes result in V(D)J recombination deficiency [4,5]. V(D)J recombination SCID is a genetically heterogeneous syndrome with the common phenotype of an early arrest of T- and/or B-maturation [6]. Cells from these patients show an increased sensitivity to ionizing radiation and alkylating agents. Mutations in the RAGs and DCLRE1C are the most common causes for V(D)J recombination SCID [7]. In clinical practice, these patients are treated with bone marrow transplantation to cure the immune defects.

NK cells are generally considered as effectors of innate immunity because they lack antigen-specific cell surface receptors [8]. Their activities are regulated by activating and inhibitory receptors and are supposed to not require RAGs and DCLRE1C. A recent study suggested that RAG deficiency impacts murine NK cells’ intrinsic responsiveness against lymphoma cells [9]. This suggests an additional role of RAGs beyond V(D)J recombination.

NK cells play an important role in the control of HCMV. Biron et al. reported an adolescent with a marked susceptibility to herpesviruses. The patient had normal immune functions, except for an extreme deficiency of NK cells. This case report suggested the importance of NK cells in controlling herpesviruses infections including active HCMV infection [10]. We have shown that NK cells can efficiently inhibit HCMV transmission in cell cultures and that NK cells degranulate and secrete IFN-γ [11,12,13]. However, the anti-HCMV role of NK cells from SCID patients with defective RAGs or DCLRE1C (RAGs^−^/DCLRE1C^−^-NK) is unknown.

## 2. Materials and Methods

### 2.1. Patients and Cells

The clinical information of these patients is summarized in Table 1. Mean age of these patients at the time of sample collection was 5.6 months. Peripheral blood mononuclear cells (PBMCs) and NK cells were isolated and cultivated as previously described [11]. NK cells were purified by magnetic negative selection (Miltenyi, Bergisch Gladbach, Germany). HCMV IgG serology of healthy donors was determined with an enzyme-linked fluorescence assay (VIDAS CMV IgG, bioMerieux, Marcy I’Etoile, France). The use of PBMCs from patients was approved by the Institutional Review Board at Ulm University. Blood samples were collected after informed consent. For mutation detection, complete coding sequences and exon/intron boundaries of the interested genes were amplified using the HotStarTaq polymerase system. PCR products were sequenced directly using the Big Dye Terminator v1.1 Cycle Sequencing Kit on an ABI PRISM 3100 Genetic Analyzer.

### 2.2. Preparation of Viral Stocks and Focal Expansion Assay

Clinical isolates E30546 and the laboratory adapted strain TB40/E were used to infect cells as previously described [11]. BAC4-UL40^repair^ reconstituted from TB40/E-BAC was propagated in human foreskin fibroblasts (HFF). For focus expansion assay, infected fibroblasts were co-cultured with a 2000-fold excess of uninfected HFF in 96-well plates. NK cells were added to the cultures at an E:T ratio of 0.25. After co-cultivation, cells were fixed with 80% acetone and immediate early antigens (IEA) were determined. The numbers of infected cells per focus were counted by ImageJ (NIH, Bethesda, MD, USA).

### 2.3. Flow Cytometry and IFN-γ Detection

The following mAbs were used: Percp-Cy5.5-anti-CD3 (UCHT1), APC-anti-CD56 (B159), PE-anti-CD57 (NK-1), PE-anti-NKp46(9E2/NKp46), PE-anti-CD158b (CH-L) and PE-anti-CD107a (H4A3) (from BD Biosciences); PE-anti-NKG2A (131411), AF700-, AF488- or purified anti-NKG2C (134591) (for the P815 coating used at 1 μg/mL, R&D Systems, Minneapolis, MN, USA); PE-Vio770-CD56 (AF12-7H3), PE-anti-CD16 (VEP13) (Miltenyi, Bergisch Gladbach, Germany); APC-anti-CD57 (HCD57; BioLegend, San Diego, CA, USA). NK cell degranulation assay was performed as previously described [12]. Cells were analyzed using FACSCalibur and sorted using FACSAria (BD Biosciences, San Jose, CA, USA). IFN-γ production was assessed in co-culture supernatants by ELISA kits (430104, Biolegend, San Diego, CA, USA).

### 2.4. Statistics

One-way-ANOVA (followed by multiple comparisons tests with LSD) was used. Results were considered significant at a *p* level of 0.05. No statistical methods were used to predetermine sample size.

## 3. Results

### 3.1. Inhibition of HCMV Transmission by NK Cells from SCID Patients with Defective RAGs or DCLRE1C (RAGs^−^/DCLRE1C^−^-NK)

**Figure 1:**
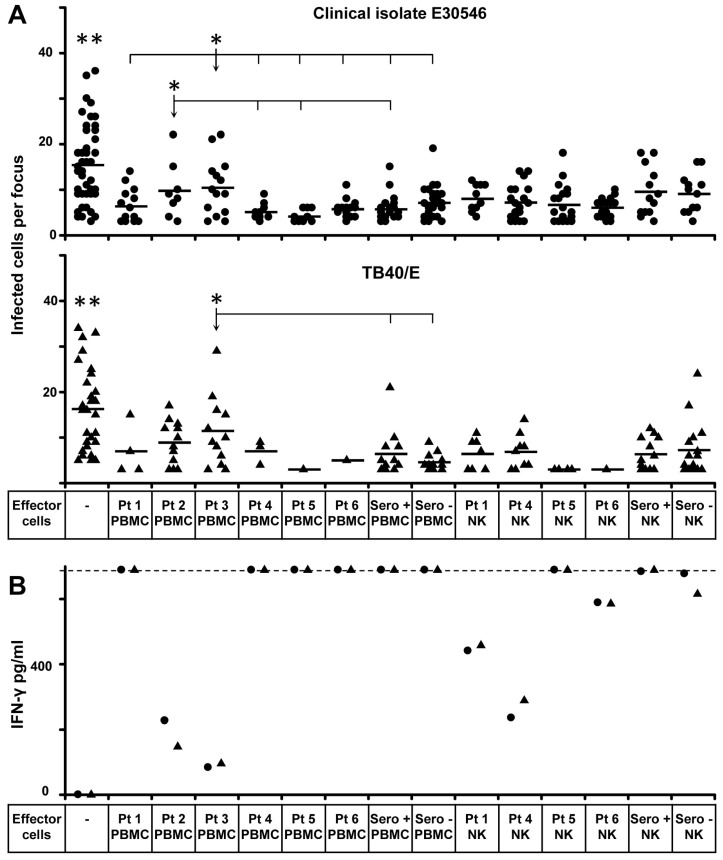
NK cells from SCID patients with defective recombination-activating genes (RAGs) or DCLRE1C inhibit HCMV transmission in fibroblasts. (**A**) Clinical isolate E30546 and TB40/E infected fibroblasts were co-cultured with 2000-fold uninfected fibroblasts for 3 days. PBMCs or purified NK cells were added to the co-cultures from the beginning. Purified NK cells were added at an E:T ratio of 0.25. The number of PBMCs were adjusted based on the percentage of NK cells to reach an E:T (NK cells:targets) ratio of 0.25. Monolayers were fixed and infected cells were monitored by HCMV IEA staining. Dots represent the number of infected cells per individual focus. Bars indicate mean values. (**B**) The supernatants of each condition were collected after 3 days post co-culture. The concentrations of IFN-γ in supernatants from E30546 infected cultures (circles) or TB40/E infected cultures (triangles) were tested by ELISA. Dashed line indicates the detection limit. * indicates *p <* 0.05 to arrow-indicated group, ** indicates *p <* 0.05 to all other groups.

By using our HCMV transmission inhibition assay [11], we firstly investigated whether RAGs^−^/DCLRE1C^−^-NK cells can inhibit the HCMV transmission in cell cultures. We chose this assay for two reasons. First, the assay provides a practical method to directly study the control of HCMV transmission and underlying mechanisms instead of measuring the activation of immune cells. Second, it requires very low amounts of NK cells, which makes functional analysis of rare immune cells possible. Since HCMV strains spread differently in cell cultures, we used the clinical HCMV isolate E30546 and the lab strain TB40/E in our study. The clinical isolate E30546 expanded strictly by cell-to-cell transmission whereas TB40/E is transmitted via cell-free virus and cell-to-cell contact [11]. We first applied PBMCs as effectors, due to the limited number of cells available from patients 2 and 3. As shown in Figure 1A, all PBMCs from RAGs^−^ or DCLRE1C^−^ SCID (Table 1) can inhibit both E30546 and TB40/E transmission between fibroblasts comparing to the condition without any effectors. In our previous studies, we found that T cells and NK cells from healthy donor PBMCs are effectors in inhibiting HCMV transmission, whereas B cells are not involved (unpublished data). Additionally, we purified NK cells from patients 1, 4, 5 and 6, and found that the NK cells can similarly inhibit the transmission of HCMV comparing to purified NK cells from healthy donors (Figure 1A). We had shown that NK cells control the HCMV transmission both via IFN-γ and by cell contact [11]. IFN-γ production could be found when using PBMCs as effectors from all patients and also with purified RAGs^−^/DCLRE1C^−^-NK cells from patients 1, 4, 5 and 6 (Figure 1B). PBMCs containing same amount of NK cells produced more IFN-γ than using purified NK cells from the same donor. This is because T cells also respond to HCMV infected cells in the same assay [14]. The IFN-γ production by purified NK cells from patients 1, 4 and 6 were lower than heathy adult controls. Furthermore, PBMCs from patients 2 and 3 secreted lower amounts of IFN-γ than PBMCs from other patients and two healthy donors. The diminished IFN-γ activities were also reflected in the degree of inhibiting virus transmission. PBMCs of patient 2 showed less inhibition of E30546 transmission than patients 4, 5 and one healthy donor. PBMCs of patient 3 showed less inhibition of E30546 transmission than patients 1, 4, 5, 6 and healthy donors with less inhibition of TB40/E transmission.

### 3.2. Phenotype of NK Cells from Defective V(D)J Recombination SCID

It has been shown that some SCID patients have leaky mutations in genes of RAG1/2 and Artemis with different amount of T cells [15]. In addition, transplacentally acquired maternal T cells are a common feature in SCID patients [16], whereas the maternal NK cells can never be found by selective HLA typing (unpublished data). Patients 1 and 2 had high amounts of maternal T cells (Table 1). Patients 5 and 6 had relatively low amounts of T cells due to leaky mutations. Notably, very limited numbers of CD56^+^T-cells could be found in our patients. Due to low amounts of B and T cells, the percentages of NK cells in lymphocytes varied from 28.25% to 88.6% in our patients, which is much higher than in healthy donors at the same age (5%) [17].

**Figure 2:**
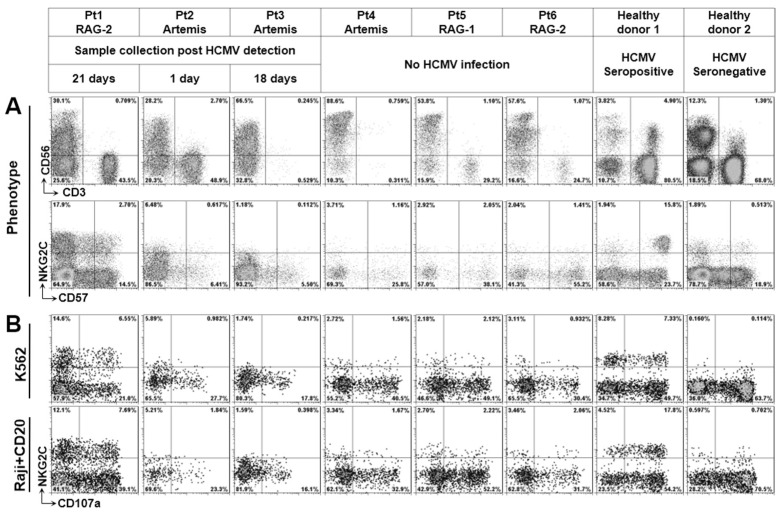
Phenotypic and functional characterization of RAGS^−^/DCLRE1C^−^-NK cells. (**A**) PBMCs were stained with CD3, CD56, CD57 and NKG2C. After gating on lymphocytes, CD3 and CD56 were used to identify NK cells. Then, the resulting expressions of NKG2C and CD57 on NK cells are shown. (**B**) PBMCs were cultured for 24h hours and afterwards co-cultured with K562 cells (E:T = 10:1) or Rituximab (CD20)-coated Raji cells (E:T = 10:1) for 5 h in the presence of anti-CD107a. After gating on lymphocytes, CD3 and CD56 were used to identify NK cells. Then, cell surface expression of NKG2C and CD107a on NK cells was assessed.

HCMV infection selectively expands NKG2C^+^ NK cells, which can be found in one-third of HCMV seropositive donors [12]. We investigated whether NKG2C^+^ NK cells can be detected in patients with RAGs or DCLRE1C deficiency. As shown in Figure 2A, patient 1 with RAG2 deficiency who was HCMV-infected indeed had NKG2C^+^ NK cells, whereas the subpopulation could not be found in other patients. We further investigated the natural cytotoxic activity and antibody-dependent cellular cytotoxic activity (ADCC) of these NK cells (Figure 2B). The degranulation of these NK cells in response to K562 cells and antibody-coated Raji cells were lower than in adult donors (except patient 5). In general, cytotoxic activity of NK cells from a fetus or cord blood is much lower than from adults [18,19]; this may be also true for our patients (mean age 5.6 months). Since these patients were treated by bone marrow transplantation to cure the fundamental immune defect, there was no chance to study the RAG- or DCLRE1C-deficient NK cells from adults. To compare the results with NK cells from age-matched healthy individuals, further studies with defined cohorts and large numbers of patients should be considered.

### 3.3. Clonal Expansion of NKG2C^+^ NK Cell in Patient 1 with RAG-2 Deficiency Post HCMV Acute Infection

**Figure 3:**
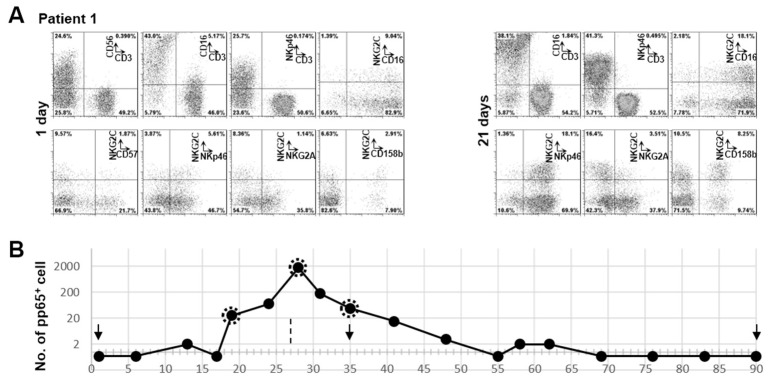
Dynamic expansion of RAGS^−^/DCLRE1C^−^-NK cells post HCMV active infection. (**A**) PBMCs from 1 day or 21 days post HCMV detection from patient 1 were stained with indicated mAb. After gating on lymphocytes, CD3/CD56, CD3/CD16 and CD3/NKp46 were used to identify NK cells. The expressions of CD57, CD16, NKp46, NKG2A and CD158b on NKG2C^+^ NK cells were analyzed after gating on CD3^−^CD56^+^ lymphocytes. (**B**) Virus detection from patient 1. The numbers of pp65 positive cells per half million of polymorphonuclear leukocytes were plotted with the days post HCMV detection. The dates of urine test are indicated with arrows. The date of bone marrow transplantation is indicated with a dashed line. The dashed circles indicate viruses that were isolated by culturing polymorphonuclear leukocytes from peripheral blood with indicator cells.

HCMV infection-induced NK cells can be phenotypically characterized by the expression of NKG2C. Since our patients cannot generate HCMV-specific T and B cells, NKG2C^+^ NK cell expansion provides an advantage to detect HCMV-specific immunity in these SCID patients. We further characterized another sample which was collected at one day post HCMV detection (Figure 2). Although we cannot define the exact time of HCMV infection in this patient, the percentage of NKG2C^+^ NK cells was nearly double after 20 days (from 11.44% to 20.6%). It had been shown that HCMV-induced NKG2C^+^ NK cells preferentially express self-specific KIRs, especially CD158b [12,20]. KIR repertoire dominance reflects NK subsets as the consequence of adaptation of the NK cell compartment to exogenous agents [21], which might be an HCMV infection. We found that the percentage of NKG2C^+^CD158b^+^ NK cells to be increased from 2.91% to 8.25% after 20 days (Figure 3A), demonstrating that NKG2C^+^ NK cells were clonally expanded after HCMV infection.

The live virus was firstly detected from urine by cell cultures (day 1). The pp65 positive polymorphonuclear leukocytes can be detected in the following 62 days and the positivity peaked at day 28. The highest amount of pp65 positive cell was 1750 per half million of polymorphonuclear leukocytes at day 28. Live virus could be isolated by culturing polymorphonuclear leukocytes from peripheral blood with indicator cells at days 19, 28 and 35. Urine cultures were negative at days 35 and 90. We captured the expansion of NKG2C^+^ NK cells (Figure 3A) with the increased viral load in the peripheral blood. Bone marrow transplantation was performed on day 27 (Figure 3B).

Of note, NKG2C is functional in this patient, as evidenced by the degranulation against NKG2C antibody coated P815 cells (data not shown). The NKG2C^+^ NK cells from patient 1 also degranulate against K562 cells and Rituximab coated RAJI cells (Figure 2B). Previously, we found that NKG2C^+^ NK cells from adults are intrinsically responsive to signaling through CD16 [12]. However, the NKG2C^+^ NK cells from patient 1 did not show a hyper-responsiveness against Rituximab-coated RAJI cells compared to NKG2C^+^ NK cells from adults. The NKG2C^+^ NK cells from this patient were mostly CD57 negative, which is also different to NKG2C^+^ NK cells from adults expressing a high level of CD57 [12]. This might be due to CD57 being poorly expressed on NK cells during the early life of individuals [18,19]. Most NKG2C^+^ NK cells from this patient were CD16 positive and NKG2A negative, similar to NKG2C^+^ NK cells from adult healthy donors [12]. Interestingly, the percentage of CD3^−^NKp46^+^ cells increased from 52.3% to 88% post HCMV detection (Figure 3A).

### 3.4. Both NKG2C^+^ and NKG2C^−^ NK Cells Are Functional to Inhibit HCMV Transmission

NKG2C^+^ NK cells can only be found in HCMV experienced individuals [22], but their exact role in the control of HCMV transmission is unknown. We and other groups have shown previously that NKG2C^+^ NK cells from healthy donors are highly responsive to HCMV infected cells in the presence of HCMV-specific antibodies [12,23], whereas they are functionally poor effectors of natural cytotoxicity. HCMV-specific antibodies could not be produced by patient 1 after HCMV infection due to the defective V(D)J recombination. Therefore, we investigated whether NKG2C^+^ NK cells themselves are functional to inhibit HCMV transmission in the absence of HCMV antibody. In individuals lacking the NKG2C gene, HCMV-induced NK cells can be detected [24], defined by downregulation of FcεR1γ and promyelocytic leukemia zinc finger protein. However, so far for functional analyses, viable HCMV-induced NK cells can only be purified by NKG2C surface staining and sorting. We did not have enough PBMCs to purify NKG2C^+^ NK cells from patient 1. Consequently, we selected a healthy donor with a similar percentage of NKG2C^+^ NK cells and expression pattern of CD158b for our functional analysis.

**Figure 4:**
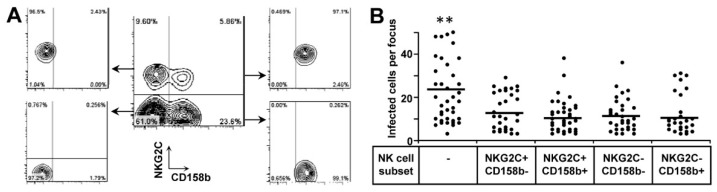
NKG2C^+^ NK cells are functional to inhibit HCMV transmission. (**A**) Gating and sorting strategy. NK cells were sorted from PBMCs based on CD3, CD56, NKG2C and CD158b expression. Initial gating on forward and side scatter was used to include lymphocytes. CD3 and CD56 were used to identify NK cells. NKG2C and CD158b were used to differentiate NK subsets. (**B**) BAC4-repUL40 infected fibroblasts were co-cultured with 2000-fold uninfected fibroblasts for 3 days. Four sorted NK subsets were added to the focus expansion assay settings at an E:T ratio of 0.25. Monolayers were fixed and infected cells were monitored by HCMV IEA staining. Dots represent mean values of infected cells per focus. Bars indicate mean values. ** indicates *p <* 0.05 to all other groups.

The HCMV glycoprotein UL40 can upregulate the cell surface expression of HLA-E which in turn is recognized by NKG2C [25,26]. It has been shown before that TB40/E carries a stop mutation in UL40 [27]. To exclude a possible effect of this mutation, we repaired the UL40 mutation in the background of BAC-4, which is derived from TB40/E. We sorted four different subpopulations of NK cells as effectors based on the expression of NKG2C and CD158b (Figure 4A). The same amount of NK cells from every subset was applied in the HCMV transmission inhibition assay. In this assay, four NK cell subsets inhibited the HCMV transmission to the same extent (Figure 4B). This indicates that NKG2C^+^ NK cells can inhibit the HCMV transmission in cell culture independent of CD158b expression.

## 4. Discussion

We provide first evidence that RAGs^−^/DCLRE1C^−^ human NK cells are functionally active against virus infections in vitro. We evaluated cells from six SCID patients in our HCMV transmission inhibition assay, which is a sensitive method to directly study the control of HCMV transmission by immune cells. PBMCs and purified NK cells from all patients inhibited the HCMV transmission in cell cultures. Although we could not compare the NK cells from age-matched healthy individuals, these results suggest a contribution of NK cells against a HCMV infection in the absence of adaptive immune cells.

RAG- and DNA-PKcs-deficient murine NK cells have two prominent features. They are hyper-responsive against YAC-1 cells at rest and exhibit diminished cellular fitness during viral infection due to defects in the repair of DNA breaks [9]. The reason for the hyper-responsiveness of these NK cells is still not clear, probably related to a more mature and active phenotype, and the authors suggested both intrinsic and extrinsic factors may contribute their functionality based on mixed chimera experiments [9].

A recent study characterized the phenotype and function of NK cells from 66 patients with RAG and non-homologous end joining gene (NHEJ) defects [28]. T cell and B cell lymphopenia were observed in the vast majority of patients and NK cell count was either normal or increased in most of them. In contrast to the RAG-deficient murine NK cells having a mature phenotype, these patients have a higher frequency of immature NK cells than healthy infants. It is not clear if the high frequency of CD56^high^ NK cells is due to RAG deficiency or a secondary effect of lacking T- and B cells. These cells are mainly NKG2A^+^ and CD57^−^. Despite the immature phenotype, the study suggested that RAGs^−^/NHEJ^−^ human NK cells express higher amount of perforin and show an enhanced degranulation activity against K562 cells in the absence of IL-2 than NK cell from healthy infants.

In our study, the percentages of NK cells in lymphocytes varied from 28.25% to 88.6%. Patients 4 and 5 have high percentages of CD56^high^ NK cells. RAGs^−^/DCLRE1C^−^ human NK cells showed a variable degranulation with a mean value of 33% against K562 cells in the presence of IL-2. We have no conclusion on the intrinsic activity of RAGs^−^/DCLRE1C^−^ human NK cells due to lack of age-matched heathy controls. In our assay, the NK cells from three of four patients secreted lower amounts of IFN-γ than NK cells from adults, however they did control the virus transmission between cells in vitro. This assay evaluates that immune cells control HCMV transmission both via IFN-γ and by cell contact [11]. We did not evaluate the direct killing of these NK cells against infected cells.

HCMV infection preferentially expands NKG2C^+^ NK cells which can be found in one-third of HCMV seropositive individuals, even in healthy donors without responsiveness of specific T cells to HCMV peptides stimulation [29]. In SCID patients with defective V(D)J somatic recombination, who cannot generate antigen-specific T and B cells, NKG2C^+^ NK cell expansion might provide a marker for the detection of an HCMV-induced immune response in some of these patients, although we cannot exclude the role of maternal T cells in the generation of NKG2C^+^ NK cells in patient 1. We suggest including phenotypical characterization of HCMV-induced NK cells in further studies in these patients for evaluation as a prognostic marker.

So far, NKG2C^+^ NK cell expansion in SCID patients post HCMV infection was reported in a patient with IL-7 receptor deficiency exhibiting a T^−^B^+^NK^+^ phenotype [30], a patient with atypical Janus kinase 3 deficiency exhibiting a T^low^B^+^NK^+^ phenotype [31] and patients with transporter associated with antigen presentation deficiency [32]. Interestingly, the NKG2C^+^ NK cell expansion in IL-7 receptor-deficient SCID reversely correlated with the level of HCMV DNA. This case report suggested that NK cells can control HCMV infection in the absence of T cells [28]. The NKG2C^+^ NK cells from JAK3 deficient patients showed a hypo-responsiveness against K562 cells and a redirected ADCC. A similar hypo-responsiveness was also observed in NKG2C^+^ NK cells with TAP deficiency. Here, we provide the new information that the clonal expansion of NKG2C^+^ NK cells after HCMV acute infection, evidenced by pp65+ polymorphonuclear leukocytes and live virus cultures, does occur in a RAG-2-deficient patient. Interestingly, 20% of CD56^+^ NK cells are NKG2C positive in healthy infants whose HCMV serostatus is not available [28]. This might due to immature NK cells, including NK cells from cord blood, having a higher percentage of NKG2C^+^CD57^–^ cells [33]. We did not observe this phenotype in our patients.

We demonstrated that purified NKG2C^+^ NK cells can inhibit the HCMV transmission in cell culture. A study using another readout is mainly in line with our findings. Chen et al. described that purified NKG2C^+^ NK cells compared to other NK cell subsets did not show any differences in controlling HCMV dissemination in vitro [34]. The role of HCMV antigens for HCMV-induced NK cells expansion has not been identified. The glycoprotein UL40 up-regulates the cell surface expression of HLA-E which in turn is recognized by NKG2C [25,26]. Recent studies suggest the peptides from UL40 can shape the HCMV-induced NK cells through NKG2C [35,36]. HCMV encodes the MHC-I homolog glycoprotein UL18 which can directly bind to NKG2C with low affinity and to LIR-1 with high affinity [37,38]. HCMV-induced NK cells also highly express LIR-1, an inhibitory receptor which contributes to NK cell education [39]. Although the role of UL18 on NK cells is still controversial in viral infection setting [40], a recent study suggested that LIR-1 polymorphisms influence post-transplant HCMV susceptibility and viral ligand UL18 binding [41]. It would be interesting to investigate whether some HCMV deletion mutants can be better controlled by NK cell subsets with our assay. This may help to define viral genes which are important for the generation and function of HCMV-induced NK cells.

## Figures and Tables

**Table 1:** Characterization of studied patients.

	Gender (f/m)	Known Mutations	Cell Count/μL			Age ^#^	
			T	B	NK		Virus Detection
Pt 1	f	RAG2: c.(572C>A); (572C>A), p.(Ser194X); (Ser194X)	1300 *	0	910	110	VI **from urine
Pt 2	m	Artemis: c.(1147C>T); (1147C>T), p.(Arg383X); (Arg383X)	283 *	0	554	122	VI ** from urine
Pt 3	m	Artemis: c.((?_-38)_246+?del); ((?_-38)_246+?del), p.(0);(0)	3	0	306	96	pp65 antigenemia
Pt 4	f	Artemis: c.((?_-38)_246+?del); ((?_-38)_246+?del), p.(0);(0)	0	0	1984	196	-
Pt 5	m	RAG1: c.(1331C>T); (1331C>T), p.(Ala444Val); (Ala444Val)	392	0	1094	285	-
Pt 6	m	RAG2: c.(475C>T); (475C>T), p.(Arg159Cys); (Arg159Cys)	198	0	276	199	-

* Maternal T cells; ^#^ age at PBMCs collection (days); ** virus isolation.
